# Analysis of the clinical efficacy and safety of anti‐PD‐1 immune checkpoint inhibitors in locally advanced nasopharyngeal cancer

**DOI:** 10.1002/cam4.7359

**Published:** 2024-07-20

**Authors:** Shuling Shi, Bingyan Li, Pengcheng Zhou, Linhui Chen, Huizhen Li, Yingyi Wang, Xiaoyu Deng, Qianqian Dang, Jingjing Wu, Boya Zha, Peihong Li, Yingjuan Zheng, Daoke Yang

**Affiliations:** ^1^ Department of Radiation Oncology The First Affiliated Hospital of Zhengzhou University Zhengzhou Henan China; ^2^ Institute of Radiotherapy and Critical Care Oncology Zhengzhou University Zhengzhou Henan China

**Keywords:** immune checkpoint inhibitors, immune‐related adverse events, nasopharyngeal cancer, PD‐1

## Abstract

**Objective:**

To analyze the efficacy and adverse effects of anti‐PD‐1 immune checkpoint inhibitors aimed at nasopharyngeal carcinoma (NPC).

**Methods:**

During the first stage of the study, using 40 patients with stage III/IVa NPC treated with anti‐PD‐1 immune checkpoint inhibitors in combination with chemoradiotherapy as a first‐line treatment (observation group) and 70 patients with NPC treated with chemoradiotherapy alone (control group). In the second stage of the study, 88 patients with NPC treated with immune checkpoint inhibitors were grouped according to the number of lines of immunotherapy, the number of times, and the types of application.

**Results:**

Observation of the short‐term effects in the first stage indicated that the objective response rate (ORR) of the observation group and the control group against primary foci of NPC was 75.0% versus 40.0%; the mortality rate of the observation group was much lower than that of the control group. The overall first‐line treatment evaluation of the observation vs. control groups were as follows: ORR (67.5% vs. 38.6%); median PFS (17.52 vs. 17.21 months); and median OS (18.68 vs. 18.14 months), respectively (*p* < 0.05). The second stage of the study had an ORR of 53.4%, and the efficacy of immunotherapy was related to staging, timing, and frequency.

**Conclusion:**

Anti‐PD‐1 immune checkpoint inhibitors combined with chemoradiotherapy as the first‐line treatment for nasopharyngeal carcinoma may improve patient outcomes significantly. Timing, frequency, and the type of immunotherapy exerted an effect on the efficacy of immunotherapy. Adverse effects that occurred during treatment were tolerable and controllable.

## 1 INTRODUCTION

The incidence of nasopharyngeal carcinoma (NPC) in southern China is as high as 70%–80% of that in China as a whole, with the incidence of men being approximately 2.6 times that of women.^1^ Radiation and chemotherapy remain the first choice treatment for NPC, while the application of intensity‐modulated radiotherapy (IMRT) has also been found to help control local lesions and prolong the overall survival (OS) of patients,^2^, ^3^ as a result of which the total 5‐year survival rate can be as high as 90%–100%. However, more than 30% of patients with either locoregional recurrence or distant metastases^4^, ^5^ continue to pose a challenge to those attempting to resolve NPC. In cases where first‐line radiotherapy and chemotherapy against NPC have already failed, the probability of back‐line treatment succeeding is poor. Although targeted treatment has reportedly exerted good curative effects on patients with NPC,^6^, ^7^ such therapy inevitably produces drug resistance,^8^ indicating the urgency for detecting new treatments that may help enhance the prognosis of patients with NPC.

With the recent advent of tumor immunotherapy, the use of immune checkpoint inhibitors (ICIs) has risen to become a research hotspot in the treatment of NPC. Immune checkpoint inhibitors are considered as a step toward “immune normalization,”^9^ wherein specific defects or dysfunctions in immune response are identified and corrected, thereby restoring lost anti‐tumor immune function. The main ICIs currently used in clinical practice are anti‐programmed death receptor 1 (PD‐1)/programmed death ligand 1 (PD‐L1) immune checkpoint inhibitors. Several studies have shown that ICIs may enhance anti‐tumor activity without enhancing normal cytotoxicity.^10^, ^11^ Current clinical application of ICIs involves the use of anti‐PD‐1/PD‐L1 immune checkpoint inhibitors. PD‐L1 is an immunosuppressive molecule expressed on T cells, B cells, dendritic cells, and tumor‐infiltrating lymphocytes.^12^ Tumor cells express PD‐L1 to recruit a large number of immune cells and cytokines, to accelerate the transformation to a immunosuppressive tumor microenvironment, eventually achieving immune escape.^13^ Thus, PD‐1/PD‐L1 immune checkpoint inhibitors are aimed at nullifying immune escape, by blocking the binding of PD‐1 on the surface of T cells to PD‐L1 on the surface of tumor cells.^14^ A large number of clinical trials have confirmed the therapeutic efficacy of anti‐PD‐1/PD‐L1 immune checkpoint inhibitors in NPC.^15^, ^16^, ^17^ Moreover, according to the Chinese Society of Clinical Oncology immunotherapy guidelines,^18^ combined therapy involving gemcitabine + cisplatin + anti‐PD‐1 immune checkpoint inhibitors has become the new standard first‐line treatment, thus signifying the entrance of NPC into the era of immunotherapy. Moreover, immune‐related adverse events (irAEs) induced by immune checkpoint inhibitors are less toxic than those induced by traditional immune‐enhancing therapies.

In the present study, we retrospectively analyzed the efficacy of ICIs in the treatment of patients with NPC, as well as the adverse aspects of their usage, in the hope of building a reference base pertaining to the application of immunotherapy to NPC in clinical practice.

## 2 MATERIALS AND METHODS

### 2.1 Clinical data

In this study, we retrospectively recruited 40 patients with stage III/IVa NPC who had been treated with anti‐PD‐1 ICIs as first‐line treatment, 48 patients with recurrent and metastatic NPC, and 70 patients with stage III/IVa NPC treated with conventional chemoradiotherapy from June 2020 to June 2023. The inclusion criteria were as follows: (i) the pathological types of undifferentiated diversification type squamous cell carcinoma; (ii) NPC patients who had received different lines of treatment with ICIs during the treatment period; (iii) those aged 18 to 70 years; (iv) a Karnofsky (KPS) score >70 before treatment; and (v) at least one measurable lesion. The exclusion criteria were as follows: (i) Patients with other types of cancer; (ii) merger related immune diseases, such as immune associated pneumonia and diabetes immune correlation; (iii) a history of severe and uncontrolled medical diseases, such as diabetes and cardiovascular disease; and (iv) pregnancy in women. This treatise was presented in two parts, with the first part being devoted to a study of the clinical efficacy as well as adverse reactions pertaining to 40 patients with stage III/IVa NPC treated with ICIs in combination with chemoradiotherapy as the first line of treatment, compared with 70 patients with NPC treated with conventional chemoradiotherapy,^19^ and the second part being dedicated to a to study of the clinical efficacy and adverse reactions of 88 NPC patients treated with ICIs, who were grouped according to the number of lines of immunotherapy, the number of times, and the types of ICIs applied. The general data of the two groups including gender (*p* = 0.591), age (*p* = 0.240), clinical stage (*p* = 0.375), T stage (*p* = 0.665), N stage (*p* = 0.852), KPS score before treatment (*p* = 0.962), smoking history (*p* = 0.934), drinking history (*p* = 0.770), diabetes history (*p* = 0.800), hypertension history (*p* = 0.849), and body mass index (BMI) (*p* = 0.686) were no significant differences (Table 1). Clinical characteristics of the 88 NPC patients treated with ICIs in the second part are shown (Table 2).

**TABLE 1:** General information of patients in both groups.

Patient characteristics	ICI first‐line treatment group (cases)	Chemoradiotherapy group (cases)	*χ* ^2^	*p*
Total cases	40	70		
Genders				
Man	31	51	0.289	0.591
Women	9	19		
Age (years)				
≥45	29	43	1.380	0.240
<45	11	27		
Clinical staging				
III	31	59	0.788	0.375
IVa	9	11		
T staging				
T1	6	10	1.575	0.665
T2	15	25		
T3	11	26		
T4	8	9		
N staging				
N0	1	3	0.791	0.852
N1	4	10		
N2	34	54		
N3	1	2		
KPS score				
≥90	33	58	0.002	0.962
<90	7	12		
BMI (kg/m^2^)				
≥18.5	35	63	0.164	0.686
<18.5	5	7		
Smoking history	10	18	0.007	0.934
Drinking history	6	12	0.085	0.770
Hypertension history	5	11	0.036	0.849
Diabetes history	2	3	0.030	0.800

**TABLE 2:** Clinical data of 88 patients with immunotherapy for nasopharyngeal carcinoma.

Clinical features	*N* (the number of cases)	The percentage
Genders		
Man	74	84.1%
Women	14	15.9%
Age (years)		
≤45	51	58.0%
>45	37	42.0%
BMI (kg/m^2^)		
≥18.5	69	78.4%
<18.5	19	21.6%
TNM staging		
III	35	39.8%
IVa	18	20.4%
IVb	35	39.8%
Immunotherapy line number		
1	40	45.5%
2	25	28.4%
≥3	23	26.1%
Treatment program		
Single ICI group	15	17.1%
ICIs with chemotherapy group	44	50.0%
ICIs with targeted group	9	10.2%
ICIs with chemotherapy and targeted group	20	22.7%
Number of ICIs		
≤5	46	52.3%
5–10	25	28.4%
≥10	17	19.3%
Types of ICIs		
Camrelizumab	44	50.0%
Toripalimab	16	18.2%
Sintilimab	17	19.3%
Tislelizumab	11	12.5%

### 2.2 Treatment

The first part of the study: the 70 chemoradiotherapy patients were subjected to radiation therapy via CT secondary positioning, application of VMAT treatment, a total radiation dosage from 66 to 70 Gy (2.0–2.12 Gy/time for a total of 30 ~ 33 times), radiation five times a week, with all patients receiving a cycle for the root of radiotherapy; these patients also received synchronous chemotherapy according to the PF scheme cisplatin 20 mg/m^2^ fluorouracil 500 mg/m^2^, amuse (5 days) and the GP scheme (gemcitabine 1000 mg/m^2^, 1, 8 days and cisplatin, 80 mg/m^2^, 1 ~ 3 days); in addition the 40 patients marked for ICI in the first‐line immunotherapy group received camrelizumab/toripalimab/sintilimab/tislelizumab 200 mg ivgtt, usually starting at the beginning of radiotherapy, for a treatment period of 21 days; the chemoradiotherapy scheme was the same as that for the control group.

The second part of the study: the 48 patients with recurrent/metastatic NPC were treated with ICIs along different treatment lines; 44 cases were treated with combination chemotherapy, with 15 receiving immunological monotherapy, 9 receiving combination targeted therapy, and 20 receiving combination chemotherapy plus targeted therapy. Chemotherapy drugs included albumin, docetaxel, gemcitabine, and pemetrexed, while targeted drugs included bevacizumab, anlotinib, and apatinib. The types of ICIs were consistent with ICIs administered to the 40 cases in the first‐line treatment immunotherapy group.

### 2.3 The evaluation of curative effect and the adverse reactions

In the first part, the short‐term effects on 110 patients who received first‐line treatment were evaluated, by assessing the curative effects of first‐line treatment on all patients. The standard for therapeutic effect evaluation was based on response evaluation criteria in solid tumors (RECIST) version 1.1,^20^ which involved the following evaluation indexes: complete response (CR), partial response (PR), stable disease (SD), progressive diseases (PD), and objective response rate (ORR). The proportion of the CR and PR indexes of the two groups of patients was calculated as (CR + PR)/(total number). For the purposes of this study, progression‐free survival (PFS) was defined as the period of time sans disease progression until death or the end of follow‐up, and overall survival (OS) was defined as the period of time until death or the end of follow‐up. Locoregional recurrence‐free survival (LRRFS): pertained to the period of time starting from treatment to the recurrence of nasopharyngeal primary tumors or cervical lymph node metastases; distant metastasis‐free survival (DMFS): pertained to the period of time starting from treatment initiation until the appearance of distant metastasis. Adverse reactions during treatment were determined using the Common Terminology Criteria Adverse Events (CTCAE), version 5.0. The patients were followed up every 3 months for 2 years after the completion of treatment, and relevant examinations such as nasopharyngeal MRI/CT, chest CT, abdominal ultrasound, nasal endoscopy, whole‐body bone scan, blood routine, liver and kidney function, and thyroid function among others were completed. Follow‐up was conducted via inpatient, outpatient or telephone contact, the follow‐up period lasting until June 2023.

### 2.4 Statistical processing

SPSS23.0 software was used for statistical analyses. Count data were expressed as the number of cases and the rate (%); general clinical data of the patients included in the study and the qualitative data of the treatment results were assessed using the chi‐square test, while survival rate was calculated using the Kaplan–Meier method; the survival rates of the groups were compared using the log‐rank test and plotted on a survival curve; the survival rate of patients was considered to be statistically significant at *p* < 0.05.

## 3 RESULTS

### 3.1 Results of the first part of the study

#### 3.1.1 Short‐term effects and assessment of the efficacy of first‐line treatment

Short‐term effects: the short‐term efficacy of first‐line treatment of nasopharyngeal primary sites and cervical lymph node metastases in 40 observation group and 70 control group participants were evaluated 3 months after treatment (Table 3). All patients had primary nasopharyngeal lesions. There were 39 cases and 67 cases with lymph node metastasis in ICIs combined with chemoradiotherapy group and chemoradiotherapy group, respectively. Evaluation of the curative effect on NPC primary foci indicated that the ORR of the observation group (75.0%; 30 cases) was significantly higher (*p* < 0.001) than that of the control group (40.0%; 28 cases). Evaluation of the curative effect on the cervical lymph node metastases indicated that the ORR of the observation group (64.1%; 25 cases) was significantly higher (*p* = 0.012) than that of the control group (38.8%; 26 cases) as shown in Table 4 Assessment of the efficacy of first‐line treatment indicated that the ORR of the observation group was significantly higher (*p* = 0.004) than that of the control group.

**TABLE 3:** Indicators of the short‐term effects in the two groups of patients.

	ICI first‐line treatment group	Radiotherapy and chemotherapy group	*χ* ^2^	*p*
Regression rate of primary foci [case (%)]				
CR rate	8 (20.0)	4 (5.7)	14.081	0.003
PR rate	22 (55.0)	24 (34.3)		
SD rate	8 (20.0)	31 (44.3)		
PD rate	2 (5.0)	11 (15.7.0)		
ORR	30 (75.0)	28 (40.0)	12.510	0.000
Regression rate of cervical lymph node (case [%])				
CR rate	5 (12.8)	4 (6.0)	7.472	0.058
PR rate	20 (51.3)	22 (32.8)		
SD rate	13 (33.3)	33 (49.3)		
PD rate	1 (2.6)	8 (11.9)		
ORR	25 (64.1)	26 (38.8)	6.319	0.012

**TABLE 4:** Assessment of the efficacy of first‐line treatment in the two groups of patients.

	ICI first‐line treatment group	Radiotherapy and chemotherapy group	*χ* ^2^	*p*
Assessment of the efficacy of first‐line treatment				
CR rate	7 (17.5)	7 (10.0)	8.546	0.036
PR rate	20 (50.0)	20 (28.6)		
SD rate	7 (17.5)	22 (31.4)		
PD rate	6 (15.0)	21 ((30.0)		
ORR	27 (67.5)	27 (38.6)	8.524	0.004

#### 3.1.2 Survival analysis

**FIGURE 1:**
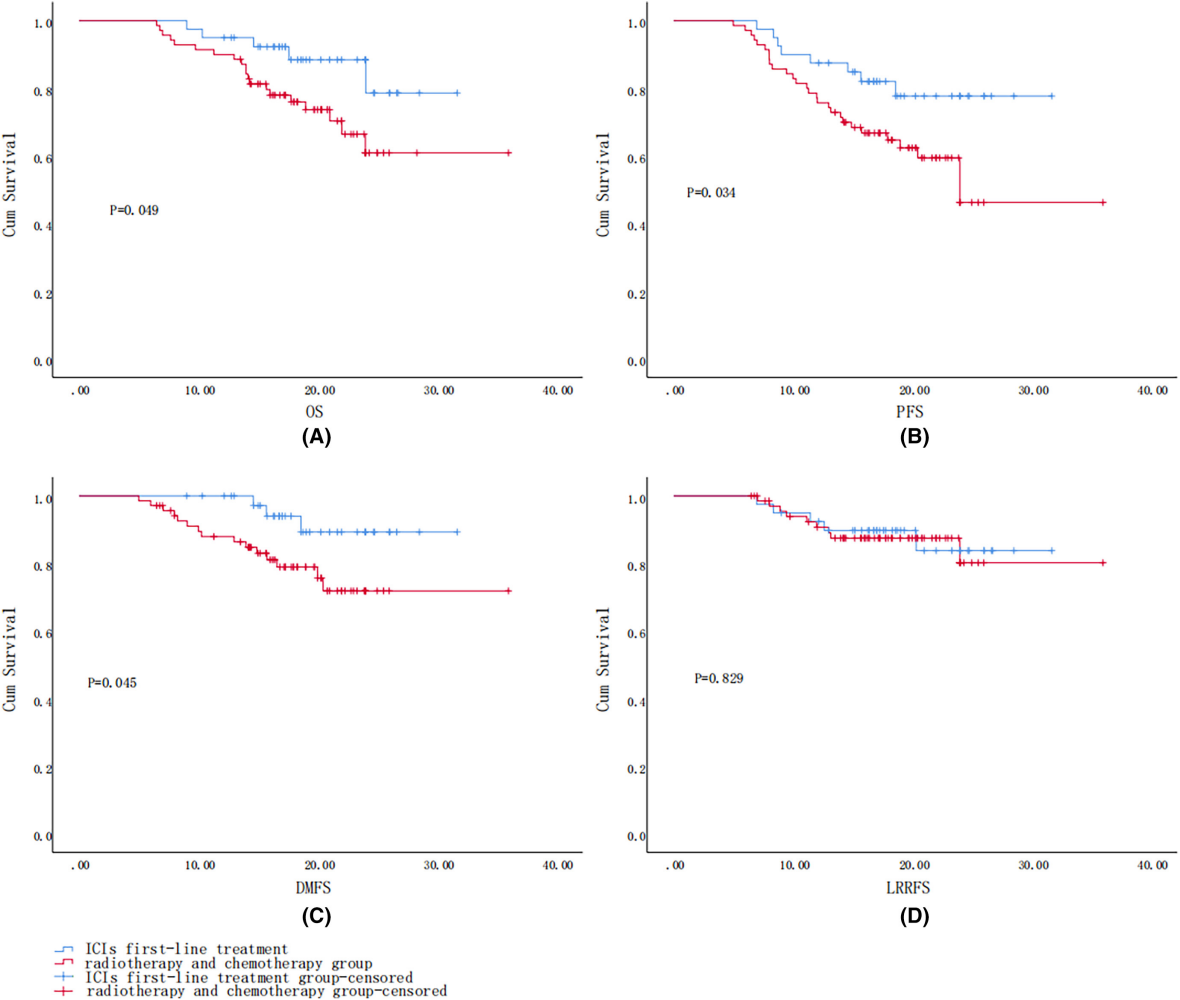
(A) Log‐rank OS curves for both groups; (B) log‐rank PFS survival curves for the two groups; (C) log‐rank DMFS survival curves for the two groups; (D) log‐rank LRRFS survival curves for both groups.

During the follow‐up period, which started in June 2020 and lasted for a total of 36 months, the median follow‐up time of the control group was 18.02 months (ranging from 6.50 to 36.0 months) and that of the observation group was 18.02 months (ranging from 9.03 to 31.70 months) 0.5 patients (12.5%) in the observation group and 20 (28.6%) in the control group died, which difference was statistically significant (*p* = 0.001). The median survival time (mOS) was 14 months (6.5–24 months), metastasis being the main cause of death. The log‐rank test was used to compare the survival curves of the two groups. The trends of OS, PFS, and DMFS curves in the first‐line ICI + chemoradiotherapy treatment group were significantly better than those in the chemoradiotherapy group (Figure 1A–C). The LRRFS of the two groups were comparatively similar, and the difference was not statistically significant (*p* = 0.829) as shown in Figure 1D.

#### 3.1.3 Adverse reactions

Adverse reactions of the two groups were mainly at the I ~ II level (Table 5). Both the first‐line ICIs + chemoradiotherapy treatment (observation group) and the chemoradiotherapy group showed different degrees of bone marrow suppression. The incidence of I ~ II level bone marrow suppression of the observation and control groups was 23 cases (57.5%) and 39 cases (55.7%), respectively, while their grade II ~ III incidences characterized by a decrease in white blood cell, neutrophil, and platelet counts were 3 cases (7.5%) and 11 cases (15.7%), respectively, with there being no statistically significant difference between the two groups. The decrease in the proportion of hemoglobin in the first‐line ICIs + chemoradiotherapy treatment group was higher than that in the chemoradiotherapy group, but no statistically significant difference was evidenced. The incidence of damage to liver function in the two groups due to injury was 45.0% and 18.6%, while the incidence of gastrointestinal reaction was 55.0%, and 80.0%, mainly manifesting as “evil” heart, vomiting, diarrhea, and constipation; the thyroid function decline rates were 60.0% and 47.1% respectively, and the differences were statistically significant. There were also some irAEs in the first‐line ICIs + chemoradiotherapy treatment group, as indicated by one case that developed Grade 2 interstitial pneumonia, causing immunotherapy to be discontinued midway, another case that presented with Grade 2 parotitis characterized by swollen parotid gland with redness, and yet another that suffered atrial fibrillation with left ventricular high voltage, high T wave, pointed, reduced ejection fraction, and higher myocardial enzyme indexes; in addition, one case presented with myocardial damage and elevated myocardial enzyme indexes, resulting in immunotherapy being suspended and support treatment being administered. In two other patients, the indicators gradually returned to normal. One case had a limb rash rated as Grade 2, two cases had a three systemic skin rash, one case presented with higher blood sugar, and one with high blood pressure.

**TABLE 5:** Comparison of adverse reactions between the two groups (cases).

ICI first‐line treatment group						Radiotherapy and chemotherapy group						
	0	I	II	III	IV	0	I	II	III	IV	*χ* ^2^	*p*
WBC decline	14	11	11	3	1	34	17	14	3	2	2.303	0.680
GRAN decline	24	10	4	2	0	49	9	3	3	1	4.245	0.341
PLT decline	33	3	3	1	0	62	5	2	1	0	1.416	0.702
HGB decline	17	19	3	1	0	40	20	7	2	1	4.662	0.324
Myelosuppression	14	12	11	3	0	20	27	12	9	2	3.986	0.408
Liver function	22	11	4	2	1	57	10	2	1	0	10.225	0.037
Creatinine	35	2	3	0	0	64	4	2	0	0	1.215	0.545
Gastrointestinal reactions	18	12	8	2	0	14	30	15	11	0	9.209	0.027
Hypothyroidism	16	15	7	2	0	37	20	7	6	0	7.995	0.046

### 3.2 Results of the second part of the study

#### 3.2.1 Relationship between clinical features and the efficacy of immunotherapy

The relationship between clinical characteristics and immunotherapy efficacy in 88 patients with NPC treated with ICIs is shown in Table 6. All patients received 1 to 23 cycles of ICIs, the median number of treatment cycles being five. The immunotherapy efficacy of ICIs in patients with NPC was not associated with gender, BMI, or the type of ICI, but rather with the stage, timing of immunotherapy, and the number of immunotherapy cycles. The chemotherapy plus targeted therapy group in the treatment regimen had the highest ORR, approximating 70.0%, whereas the group targeted with combination treatment showed the worst efficacy, the difference being insignificant (*p* = 0.737). Among the four immune checkpoint inhibitors, tislelizumab elicited the highest ORR rate (63.6%), with camrelizumab, which was used the highest number of times, eliciting the second highest (56.8%), while sintilimab had the highest PD rate (47.0%), although the difference was not statistically significant (*p* = 0.213). The ORR of first‐line application of immunotherapy was higher than that of second‐line, the third‐line, or above (*p* = 0.012); the higher the number of immunotherapy treatments, the higher the ORR, with the best efficacy being shown against NPC patients who had used immunotherapy ≥10 times (*p* = 0.044); the earlier the staging of the TNM stage, the higher the ORR rate.

**TABLE 6:** Relationship between clinical characteristics and immunotherapy efficacy in 88 NPC patients.

Clinical features	CR9	PR38	SD20	PD21	*p*
Genders					
Man	8	31	16	19	0.356
Women	1	7	4	2	
BMI (kg/m^2^)					
≥18.5	8	28	16	17	0.754
<18.5	1	10	4	4	
TNM staging					
III	7	17	5	6	0.021
IVa	2	7	6	3	
IVb	0	14	9	12	
Immunotherapy line number					
1	7	20	7	6	0.012
2	2	10	9	4	
≥3	0	8	4	11	
Treatment program					
Single ICI group	1	7	3	4	0.737
ICIs with chemotherapy group	5	17	12	10	
ICIs with targeted group	1	2	2	4	
ICIs with chemotherapy and targeted group	2	12	3	3	
Number of ICIs					
≤5次	3	15	12	16	0.044
5 ~ 10次	2	13	6	4	
≥10次	4	10	2	1	
Types of ICIs					
Camrelizumab	5	20	14	5	0.213
Toripalimab	2	6	3	5	
Sintilimab	1	6	2	8	
Tislelizumab	1	6	1	3	

#### 3.2.2 Survival analysis

**FIGURE 2:**
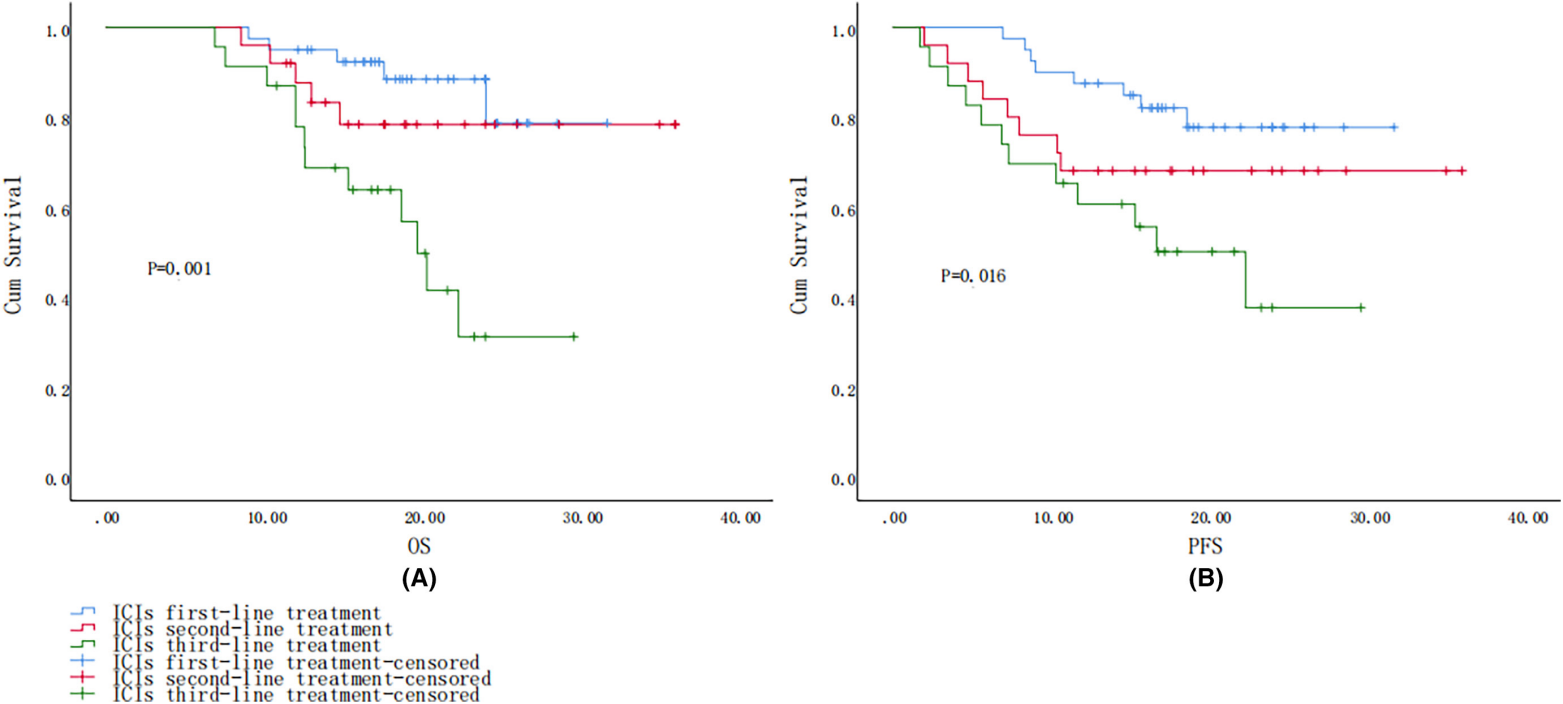
(A) Log‐rank OS curves for different lines of immunotherapy; (B) log‐rank PFS survival curves for different lines of immunotherapy.

**FIGURE 3:**
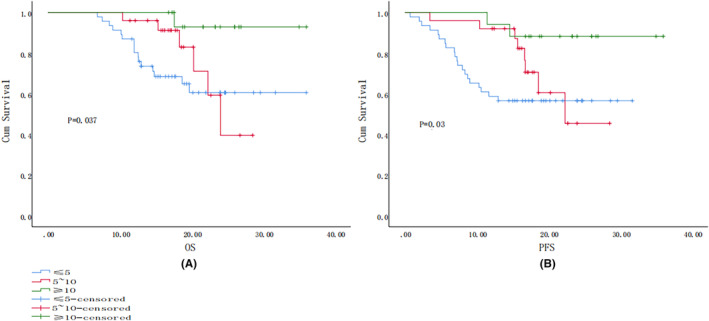
(A) Log‐rank OS curves for different numbers of immunotherapy treatments; (B) log‐rank PFS survival curves for different numbers of immunotherapy treatments.

**FIGURE 4:**
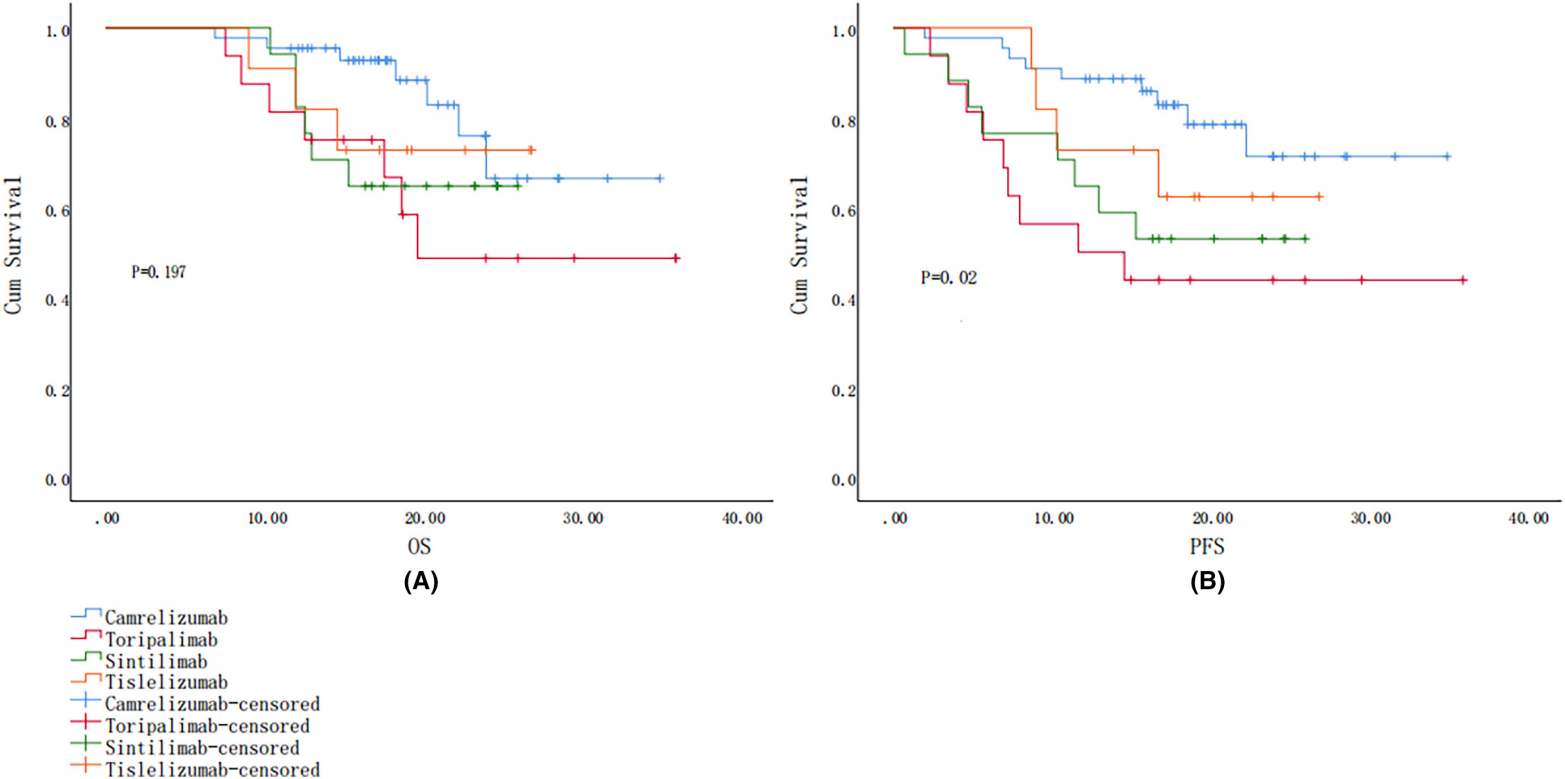
(A) Log‐rank OS curve for ICI types; (B) ICI type log‐rank PFS survival curves.

The median follow‐up time for all patients was 17.89 months (range 6.90–36.0 months). Of the 88 patients, 22 died, 5 (12.5%) during the first‐line treatment with ICIs, 5 (20.0%) in the second‐line treatment group, and 12 (52.2%) in the third‐line and above treatment group, the difference being statistically significant (*P* < 0.05), 18 (20.3%) in men and 4 (28.6%) in women, 7 (20.0%) in stage III, 6 (33.3%) in IVa, and 9 (25.7%) in IVb. The difference was not statistically significant (*p* > 0.05). The OS for all patients was 17.885 months (6.9–36 months). Survival curves for different treatment lines are depicted (Figure 2A), with the best survival being shown by the first‐line treatment, with a statistically significant difference (*p* = 0.001). Overall, 28 cases (31.8%) showed PFS, including 8 (20.0%) in the first‐line treatment group, 8 (32.0%) in the second‐line treatment group, and 12 (52.1%) in the third‐line and above treatments group the trends in the PFS of the three are shown (Figure 2B). The trend in PFS in the first‐line treatment groups was better, while the PFS in the third‐line treatment group was worse, with a statistically significant difference (*p* = 0.016). In regard to the number of treatments grouped to make a survival curve graph, we found that the higher the number of times their OS, the better the PFS trend, as shown in Figure 3A,B, the difference being statistically significant. Survival graphs corresponding to the type of ICIs indicated that the OS profile of camrelizumab was better (Figure 4A), although the difference was not statistically significant (*p* = 0.197). The trend in PFS was better for camrelizumab and worse for toripalimab (Figure 4B), with the difference being statistically significant (*p* = 0.02).

#### 3.2.3 Adverse reaction

Immunotherapy was suspended in 4 of 88 patients due to immune‐related adverse reactions. Among them, the incidence of leukocyte decline, neutrophil decline, platelet decline, gastrointestinal reaction, thyroid function decline, and rashes was higher in the ICI first‐line treatment group, but the difference was not statistically significant. The incidence of hemoglobin decline and hepatic function injury in the ICI first‐line treatment group was significantly higher (*p* < 0.05) as shown in Table 7. The irAEs seen in 48 patients with recurrent/metastatic NPC, included one with grade I generalized rash, one with grade II generalized rash, one with Grade 2 obstructive pneumonia accompanied by cough and chest tightness, one with lower limb edema, one with abnormal cortisol function, and one with proteinuria.

**TABLE 7:** Incidence of adverse effects of immunotherapy in 88 cases.

ICI first‐line treatment group						ICI recurrent metastatic treatment group						
	0	I	II	III	IV	0	I	II	III	IV	*χ* ^2^	*p*
WBC decline	14	11	11	3	1	24	10	12	2	0	3.613	0.461
GRAN decline	24	10	4	2	0	34	8	4	2	0	1.227	0.746
PLT decline	33	3	3	1	0	42	4	2	0	0	2.085	0.555
HGB decline	17	19	3	1	0	26	12	10	0	0	8.127	0.043
Liver function	22	11	4	2	1	39	7	2	0	0	9.808	0.044
Gastrointestinal reactions	18	12	8	2	0	24	16	4	4	0	2.744	0.433
Hypothyroidism	16	15	7	2	0	24	14	8	2	0	0.984	0.805
Rash	37	0	1	2	0	46	1	1	0	0	4.408	0.221

## 4 DISCUSSION

NPC is highly sensitive to radiation, and thus, radiotherapy and chemotherapy continue to remain as the first choice of treatment for NPC.^18^, ^21^ However, due to early sites of the disease being relatively hidden, initial symptoms often go unnoticed and the majority of patients are in the middle to late stages of the disease when they are diagnosed for the first time.^22^, ^23^ In addition to radiotherapy and chemotherapy, targeted therapy for patients with NPC has also introduced more choice, generally when using radiotherapy and chemotherapy, or both in combination with immunotherapy (not alone) to treat NPC.^24^ Currently, the main clinical application of targeted therapy drugs that are mainly used in clinical applications is the VEGFR inhibitors, nimotuzumab and bevacizumab, and the TKI inhibitors, apatinib and afatinib.^6^, ^7^ However, the use of such targeted therapy‐based drugs leads to issues, such as drug resistance and adverse reactions, that need to be resolved. ICIs have emerged as the new research hotspot in NPC related therapy. ICIs have been widely used in therapeutic procedures aimed at many malignant tumors, such as lung cancer, lymphoma, and melanoma.^25^, ^26^, ^27^ Many clinical trials^28^, ^29^ have proved the effectiveness of anti‐PD‐1/PD‐L1 ICIs against NPC, and treatment involving radiotherapy and chemotherapy combined with anti‐PD‐1/PD‐L1 ICIs has come to be considered as the first‐line treatment option for NPC, thereby marking the official entrance of NPC into the era of immunotherapy.

In the first part of this study, the efficacy and adverse effects exerted by a first‐line treatment for locally advanced nasopharyngeal carcinoma involving anti‐PD‐1 inhibitors combined with radiotherapy and chemotherapy were analyzed. The results indicated that the short‐term efficacy and first‐line efficacy of ICIs combined with chemoradiotherapy group were much higher than those of chemoradiotherapy group.

Survival curves indicated that the OS, PFS, and DMFS curves of the first‐line ICI treatment group were significantly better than those of the chemoradiotherapy group.

Thus, the efficacy and survival results of the first‐line treatment involving ICIs combined with chemoradiotherapy were far better than that of traditional chemoradiotherapy. At present, patients with locally advanced nasopharyngeal carcinoma are mainly treated with simultaneous chemoradiotherapy, and there are very few studies on the application of ICIs as first‐line treatment. However, even if patients with middle and advanced stages are treated with simultaneous chemoradiotherapy, the risk of subsequent local recurrence or metastasis is still greater. Therefore, this study provides a reference for the clinical application of ICI first‐line treatment in patients with locally advanced nasopharyngeal carcinoma. However, ICIs, used under clinical conditions, are not eligible for coverage under the category of medical insurance, because it is difficult to clearly predict their curative effects, as these may require further follow‐up studies; thus, their time as a first‐line treatment for NPC may be brief. The second part of the study investigated the relationship between the efficacy of immunotherapy on patients with NPC and their clinical characteristics. The results indicated that the earlier the clinical stage, the better the efficacy, and that the ORR of first‐line immunotherapy for locally advanced nasopharyngeal carcinoma was higher than that of the second‐line, the third‐line, or above. Moreover, the trends shown by the OS and PFS resulting from first‐line treatment were significantly better than those of the other therapeutic lines, suggesting that the therapeutic value of immunotherapy as a first‐line treatment for NPC may be greater. However, this result may also be related to the stage of the patients, the stage of the first‐line treatment patients is earlier, while the second‐ and third‐line patients are recurrent or metastatic patients, so the prognosis is relatively poor.

Analysis of the relationship between the number of immunotherapy sessions and efficacy indicated that the higher the number of sessions, the better the efficacy, in addition to which, the survival curves also showed that the higher the number of sessions, the better the trends shown by OS and PFS, with the differences being statistically significant. However, it cannot be ruled out that termination of immunotherapy due to the adverse effects exerted by anti‐PD inhibitors may have led to this result, which contention may need to be verified via further experimentation. However, studies have demonstrated a relative reduction in the severity of irAEs with repeated use of anti‐PD‐1/PD‐L1 inhibitors.^30^ It provides viable justification for increasing the number of immunotherapy sessions under clinical conditions. In addition, comparisons between different treatment regimens have indicated that ICIs combined with chemotherapy and targeted agents had the highest ORR, although these findings lacked statistical significance. However, an experimental study which compared the efficacy of camrelizumab combined with chemotherapy with that of camrelizumab monotherapy^31^ indicated that the former group showed an ORR of 90.9% as opposed to the 34.1% shown by the latter. These results illustrated that the efficacy of an ICI alone would be far inferior to that of an ICI combined with chemotherapy, while another study contended that the relevant theoretical concept underlying such findings may be that radiotherapy, chemotherapy, and targeted therapies may affect the function of immune cells in the tumor microenvironment, leading to the better therapeutic efficacy that is obtained via combination with anti‐PD‐1/PD‐L1 ICIs.^32^, ^33^, ^34^ In addition, radiotherapy may affect almost the entire process of immunotherapy, including sensitization and activation of immune cells, antigen loss, presentation of cancer cells, release of inflammatory factors, and regulation of the microenvironment.^35^, ^36^, ^37^ Yu et al^38^ proposed the concept of “Radscopal effect,” which contends that low‐dose radiotherapy is more conducive to the regulation of the immune microenvironment, and that it may reduce the immunosuppression caused by high‐dose radiotherapy, thereby providing a theoretical basis for combining radiotherapy with immunotherapy more effectively. The survival curves pertaining to the efficacy of different types of ICIs on NPC showed that the PFS trend of camrelizumab was better, while that of toripalimab was worse, thereby providing a reference for the clinical use of drugs for NPC patients.

Safety assessments conducted during the first part of the study indicated that the adverse reactions most frequently occurring in both groups were hematological toxicities, such as neutropenia and hemoglobin reduction, the incidence rates of which were similar, suggesting that immunotherapy had not increased the incidence of these adverse reactions. However, the incidence of liver function impairment and hypothyroidism in the immunotherapy group was higher, while the incidence of gastrointestinal reactions was higher in the chemoradiotherapy group. In addition to the above adverse reactions, irAEs observed in the immunotherapy group were interstitial pneumonitis, myocardial injury, cardiac arrhythmia, mumps, elevated blood glucose, and elevated blood pressure. The second part of the study compared the adverse effects of 40 patients who received first‐line immunological treatment with those of 48 patients with recurrent/metastatic NPC, and the results showed that the incidence of hepatotoxicity, gastrointestinal reactions, hypothyroidism, and hepatic impairment were higher in the first‐line treatment with ICI group than those in the recurrent/metastatic treatment treated with ICI group. The incidence of IrAEs in the recurrent/metastatic group was lower than that in the first‐line treatment group, which may be attributed to the differences between tumor microenvironments, and the strengths of the anti‐tumor effects. These adverse effects were basically grades I–II, and most of the side effects were well tolerated and controlled, indicating the feasibility of clinically applying immunotherapy. However, according to some studies some patients have experienced serious life‐threatening events following the use of ICIs.^39^, ^40^ Some studies have reported that the deaths associated with anti‐PD‐1/PD‐L1 were usually caused by pneumonia and hepatitis.^41^ In addition to the adverse effects seen in this study,^42^ there have been many clinical studies of irAEs, including colitis, lupus, adrenal gland abnormalities, hyperthyroidism, and peripheral neuropathy.

Some studies have shown that accurate predictive markers may be used to screen populations that may benefit from immunotherapy, in order to individualize clinical treatments, and to reduce drug resistance and adverse effects. PD‐L1 is currently a well‐established predictive marker used in clinical practice. Girault found that melanoma patients with high expression levels of PD‐1 and PD‐L1 treated with anti‐PD‐1 inhibitors experienced longer OS^43^; tumor mutational burden (TMB), which is another hotspot in the determination of predictive markers for immunotherapy, was found to be associated with the risk of tumor recurrence and the efficacy of oncology treatment.^44^, ^45^ Xu investigated the prognostic relationship between EBV DNA and the efficacy of anti‐PD‐1 immune checkpoint inhibitors against NPC^46^ and found a positive correlation between the two, suggesting that plasma EBV DNA may play a prognostic role in the detection of disease progression in NPC patients treated with ICIs, as well as microsatellite instability and tumor lymphocyte infiltration in further study.

In addition to the screening of immunotherapy using precise prediction markers, many studies have attempted to enhance the efficacy of immunotherapy by altering treatment regimens. Some studies which enhanced immune response by using two ICIs found that any enhancement in the efficacy of immunotherapy was accompanied by an increased incidence in adverse effects.^47^, ^48^ On the basis of such assessments of therapeutic effects, some studies have proposed the concept of using novel bispecific antibodies to target PD‐L1 and CTLA‐4 immune checkpoints in order to produce synergistic anti‐tumor effects. Ma^35^ conducted the first phase I trial investigating the role of a novel bispecific antibody in solid tumors, and the results showed that such antibodies may improve the therapeutic efficacy of the tumors, prolong survival, lower the incidence of adverse reactions, and improve tolerability, particularly in NPC patients. Thus, it appears to be one of the most cutting‐edge and promising strategies that could be utilized to improve the efficacy of immunotherapy by attenuating irAEs. In addition, it has been suggested that hyperthermia may be applied to reduce the number of immunosuppressive cells, such as Treg and TH17, in the tumor microenvironment and upregulate PD‐L1 expression to enhance a low response rate and lower the incidence of irAEs due to ICIs.^49^, ^50^

This study was affected by certain limitations, such as the use of a single‐center, small‐sample size, and the retrospective nature of the study. Thus, it may be necessary to further verify our conclusions via a multicenter, large‐sample prospective study; the second part of the study used a variety of immune checkpoint inhibitors, making it impossible to specifically evaluate the efficacy of each individual anti‐PD‐1 inhibitor; in addition, a variety of chemotherapeutic regimens were tested using small‐sample sizes; all these factors may impact the results of the experiments, indicating that further studies using larger sample sizes may be warranted.

## 5 CONCLUSION

In summary, therapeutic efficacy against NPC was significantly improved by a treatment involving ICIs combined with radiotherapy and chemotherapy, as compared to the chemoradiotherapy alone group, in addition to which the combined treatment did not significantly increase toxic side effects, making the resultant irAEs tolerable and controllable. Meanwhile, the efficacy of the first‐line treatment involving ICIs was much higher than that of the recurrence/metastasis treatment group, with the number and type of ICIs exerting a considerable therapeutic effect. These findings indicated the feasibility of using anti‐PD‐1 immunosuppressant therapy for NPC at an earlier stage and for a longer period of time under clinical conditions. However, clinical trials involving more samples of larger sizes are felt to be needed in order to confirm the many benefits conferred on NPC patients by ICIs.

## AUTHOR CONTRIBUTIONS

**Shuling Shi:** Writing – original draft (lead). **Bingyan Li:** Writing – review and editing (equal). **Pengcheng Zhou:** Conceptualization (equal). **Linhui Chen:** Investigation (equal). **Huizhen Li:** Formal analysis (equal). **Yingyi Wang:** Data curation (equal). **Xiaoyu Deng:** Project administration (equal). **Qianqian Dang:** Visualization (equal). **Jingjing Wu:** Conceptualization (equal). **Boya Zha:** Investigation (equal). **Peihong Li:** Supervision (equal). **Yingjuan Zheng:** Methodology (equal). **Daoke Yang:** Resources (equal).

## FUNDING INFORMATION

This study was supported by Provincial‐Ministry Joint Project of Medical Science and Technology Tackling Programme in Henan Province (SB201901037).

## CONFLICT OF INTEREST STATEMENT

All authors declare no conflict of interest.

## ETHICS STATEMENT

The authors are accountable for all aspects of the work in ensuring that questions related to the accuracy or integrity of any part of the work are appropriately investigated and resolved. The study was approved by the Ethics Committee of the First Affiliated Hospital of Zhengzhou University. Ethical number: 2022‐KY‐0925‐001.

## CONSENT FOR PUBLICATION

Exemption was granted by the Ethics Committee.

## Data Availability

Data sharing is not applicable to this article as no new data were created or analyzed in this study.

## References

[1] Sung H , Ferlay J , Siegel RL , et al. Global cancer statistics 2020: GLOBOCAN estimates of incidence and mortality worldwide for 36 cancers in 185 countries. CA Cancer J Clin. 2021;71(3):209‐249. 33538338 10.3322/caac.21660

[2] You R , Liu Y‐P , Huang P‐Y , et al. Efficacy and safety of locoregional radiotherapy with chemotherapy vs. chemotherapy alone in De Novo metastatic nasopharyngeal carcinoma: a multicenter phase 3 randomized clinical trial. JAMA Oncol. 2020;6(9):1345‐1352. 32701129 10.1001/jamaoncol.2020.1808 PMC7378870

[3] Tang L‐L , Guo R , Zhang N , et al. Effect of radiotherapy alone vs. radiotherapy with concurrent chemoradiotherapy on survival without disease relapse in patients with low‐risk nasopharyngeal carcinoma: a randomized clinical trial. JAMA. 2022;328(8):728‐736. 35997729 10.1001/jama.2022.13997 PMC9399866

[4] Tsang J , Lee VHF , Kwong DLW . Novel therapy for nasopharyngeal carcinoma—where are we. Oral Oncol. 2014;50(9):798‐801. 24462373 10.1016/j.oraloncology.2014.01.002

[5] Lee AWM , Ng WT , Chan YH , Sze H , Chan C , Lam TH . The battle against nasopharyngeal cancer. Radiother Oncol. 2012;104(3):272‐278. 22938727 10.1016/j.radonc.2012.08.001

[6] Zhao C , Miao J , Shen G , et al. Anti‐epidermal growth factor receptor (EGFR) monoclonal antibody combined with cisplatin and 5‐fluorouracil in patients with metastatic nasopharyngeal carcinoma after radical radiotherapy: a multicentre, open‐label, phase II clinical trial. Ann Oncol. 2019;30(4):637‐643. 30689735 10.1093/annonc/mdz020

[7] Ruan X , Liang J , Pan Y , et al. Apatinib for the treatment of metastatic or locoregionally recurrent nasopharyngeal carcinoma after failure of chemotherapy: a multicenter, single‐arm, prospective phase 2 study. Cancer. 2021;127(17):3163‐3171. 34043812 10.1002/cncr.33626

[8] Lung HL , Kan R , Chau WY , et al. The anti‐tumor function of the IKK inhibitor PS1145 and high levels of p65 and KLF4 are associated with the drug resistance in nasopharyngeal carcinoma cells. Sci Rep. 2019;9(1):12064. 31427673 10.1038/s41598-019-48590-7 PMC6700134

[9] Sanmamed MF , Chen L . A paradigm shift in cancer immunotherapy: from enhancement to normalization. Cell. 2018;175(2):313‐326. 30290139 10.1016/j.cell.2018.09.035 PMC6538253

[10] Ulrich BC . Clonal hematopoiesis leading to AITL and NPM1 ‐mutated AML. N Engl J Med. 2018;379(22):2184‐2185. 10.1056/NEJMc1813168 30499647

[11] Ascierto PA , Del Vecchio M , Mandalá M , et al. Adjuvant nivolumab versus ipilimumab in resected stage IIIB–C and stage IV melanoma (CheckMate 238): 4‐year results from a multicentre, double‐blind, randomised, controlled, phase 3 trial. Lancet Oncol. 2020;21(11):1465‐1477. 32961119 10.1016/S1470-2045(20)30494-0

[12] Liu Y , Wang Y , Yang Y , et al. Emerging phagocytosis checkpoints in cancer immunotherapy. Signal Transduct Target Ther. 2023;8(1):104. 36882399 10.1038/s41392-023-01365-z PMC9990587

[13] Jhunjhunwala S , Hammer C , Delamarre L . Antigen presentation in cancer: insights into tumour immunogenicity and immune evasion. Nat Rev Cancer. 2021;21(5):298‐312. 33750922 10.1038/s41568-021-00339-z

[14] Yi M , Niu M , Xu L , Luo S , Wu K . Regulation of PD‐L1 expression in the tumor microenvironment. J Hematol Oncol. 2021;14(1):10. 33413496 10.1186/s13045-020-01027-5 PMC7792099

[15] Jung HA , Park KU , Cho S , et al. A phase II study of nivolumab plus gemcitabine in patients with recurrent or metastatic nasopharyngeal carcinoma (KCSG HN17–11). Clin Cancer Res. 2022;28(19):4240‐4247. 35819451 10.1158/1078-0432.CCR-22-1238

[16] Yang Y , Qu S , Li J , et al. Camrelizumab versus placebo in combination with gemcitabine and cisplatin as first‐line treatment for recurrent or metastatic nasopharyngeal carcinoma (CAPTAIN‐1st): a multicentre, randomised, double‐blind, phase 3 trial. Lancet Oncol. 2021;22(8):1162‐1174. 34174189 10.1016/S1470-2045(21)00302-8

[17] Mai H‐Q , Chen Q‐Y , Chen D , et al. Toripalimab or placebo plus chemotherapy as first‐line treatment in advanced nasopharyngeal carcinoma: a multicenter randomized phase 3 trial. Nat Med. 2021;27(9):1536‐1543. 34341578 10.1038/s41591-021-01444-0

[18] Tang L , Chen Y , Chen C , et al. The Chinese Society of Clinical Oncology (CSCO) clinical guidelines for the diagnosis and treatment of nasopharyngeal carcinoma. Cancer Commun. 2021;41(11):1195‐1227. 10.1002/cac2.12218 PMC8626602 34699681

[19] Pfister DG , Spencer S , Adelstein D , et al. Head and neck cancers, version 2.2020, NCCN clinical practice guidelines in oncology. J Natl Compr Cancer Netw. 2020;18(7):873‐898. 10.6004/jnccn.2020.0031 32634781

[20] Eisenhauer EA , Therasse P , Bogaerts J , et al. New response evaluation criteria in solid tumours: revised RECIST guideline (version 1.1). Eur J Cancer. 2009;45(2):228‐247. 19097774 10.1016/j.ejca.2008.10.026

[21] Chen YP , Chan ATC , Le QT , et al. Nasopharyngeal carcinoma. Lancet. 2019;394(10192):64‐80. 31178151 10.1016/S0140-6736(19)30956-0

[22] Pan JJ , Ng WT , Zong JF , et al. Proposal for the 8th edition of the AJCC/UICC staging system for nasopharyngeal cancer in the era of intensity‐modulated radiotherapy. Cancer. 2016;122(4):546‐558. 26588425 10.1002/cncr.29795 PMC4968037

[23] Lee AWM , Ng WT , Chan LK , et al. The strength/weakness of the AJCC/UICC staging system (7th edition) for nasopharyngeal cancer and suggestions for future improvement. Oral Oncol. 2012;48(10):1007‐1013. 22525607 10.1016/j.oraloncology.2012.03.022

[24] Kang Y , He W , Ren C , et al. Advances in targeted therapy mainly based on signal pathways for nasopharyngeal carcinoma. Signal Transduct Target Ther. 2020;5(1):245. 33093441 10.1038/s41392-020-00340-2 PMC7582884

[25] Sezer A , Kilickap S , Gümüş M , et al. Cemiplimab monotherapy for first‐line treatment of advanced non‐small‐cell lung cancer with PD‐L1 of at least 50%: a multicentre, open‐label, global, phase 3, randomised, controlled trial. Lancet. 2021;397(10274):592‐604. 33581821 10.1016/S0140-6736(21)00228-2

[26] Manos K , Chong G , Keane C , et al. Immune priming with avelumab and rituximab prior to R‐CHOP in diffuse large B‐cell lymphoma: the phase II AvR‐CHOP study. Leukemia. 2023;37(5):1092‐1102. 36906715 10.1038/s41375-023-01863-7

[27] Li S , Yu W , Xie F , et al. Neoadjuvant therapy with immune checkpoint blockade, antiangiogenesis, and chemotherapy for locally advanced gastric cancer. Nat Commun. 2023;14(1):8. 36596787 10.1038/s41467-022-35431-x PMC9810618

[28] Fang W , Yang Y , Ma Y , et al. Camrelizumab (SHR‐1210) alone or in combination with gemcitabine plus cisplatin for nasopharyngeal carcinoma: results from two single‐arm, phase 1 trials. Lancet Oncol. 2018;19(10):1338‐1350. 30213452 10.1016/S1470-2045(18)30495-9

[29] Lu N , Jiang Y‐F , Xia W‐X , et al. Efficacy and safety of sintilimab plus bevacizumab in metastatic nasopharyngeal carcinoma after failure of platinum‐based chemotherapy: an open‐label phase 2 study. eClinicalMedicine. 2023;62:102136. 37593221 10.1016/j.eclinm.2023.102136 PMC10430191

[30] Simonaggio A , Michot JM , Voisin AL , et al. Evaluation of readministration of immune checkpoint inhibitors after immune‐related adverse events in patients with cancer. JAMA Oncol. 2019;5(9):1310‐1317. 31169866 10.1001/jamaoncol.2019.1022 PMC6555478

[31] Lv J‐W , Li J‐Y , Luo L‐N , Wang ZX , Chen YP . Comparative safety and efficacy of anti‐PD‐1 monotherapy, chemotherapy alone, and their combination therapy in advanced nasopharyngeal carcinoma: findings from recent advances in landmark trials. J Immunother Cancer. 2019;7(1):159. 31238988 10.1186/s40425-019-0636-7 PMC6593483

[32] Lv J , Wei Y , Yin J‐H , et al. The tumor immune microenvironment of nasopharyngeal carcinoma after gemcitabine plus cisplatin treatment. Nat Med. 2023;29(6):1424‐1436. 37280275 10.1038/s41591-023-02369-6

[33] Yi M , Zheng X , Niu M , Zhu S , Ge H , Wu K . Combination strategies with PD‐1/PD‐L1 blockade: current advances and future directions. Mol Cancer. 2022;21(1):28. 35062949 10.1186/s12943-021-01489-2 PMC8780712

[34] Li J‐Y , Chen Y‐P , Li Y‐Q , Liu N , Ma J . Chemotherapeutic and targeted agents can modulate the tumor microenvironment and increase the efficacy of immune checkpoint blockades. Mol Cancer. 2021;20(1):27. 33541368 10.1186/s12943-021-01317-7 PMC7863268

[35] Ma Y , Xue J , Zhao Y , et al. Phase I trial of KN046, a novel bispecific antibody targeting PD‐L1 and CTLA‐4 in patients with advanced solid tumors. J Immunother Cancer. 2023;11(6):e006654. 37263673 10.1136/jitc-2022-006654 PMC10254625

[36] Lin W , Xu Y , Chen X , et al. Radiation‐induced small extracellular vesicles as “carriages” promote tumor antigen release and trigger antitumor immunity. Theranostics. 2020;10(11):4871‐4884. 32308755 10.7150/thno.43539 PMC7163438

[37] Sharabi AB , Nirschl CJ , Kochel CM , et al. Stereotactic radiation therapy augments antigen‐specific PD‐1–mediated antitumor immune responses via cross‐presentation of tumor antigen. Cancer Immunol Res. 2015;3(4):345‐355. 25527358 10.1158/2326-6066.CIR-14-0196 PMC4390444

[38] Zhang Z , Liu X , Chen D , Yu J . Radiotherapy combined with immunotherapy: the dawn of cancer treatment. Signal Transduct Target Ther. 2022;7(1):258. 35906199 10.1038/s41392-022-01102-y PMC9338328

[39] Richter MD , Crowson C , Kottschade LA , Finnes HD , Markovic SN , Thanarajasingam U . Rheumatic syndromes associated with immune checkpoint inhibitors: a single‐center cohort of sixty‐one patients. Arthritis Rheum. 2019;71(3):468‐475. 10.1002/art.40745 30281202

[40] Correction: comprehensive meta‐analysis of key immune‐related adverse events from CTLA‐4 and PD‐1/PD‐L1 inhibitors in cancer patients. Cancer Immunol Res. 2018;6(4):498‐499. 29559478 10.1158/2326-6066.CIR-18-0078

[41] Wang DY , Salem J‐E , Cohen JV , et al. Fatal toxic effects associated with immune checkpoint inhibitors: a systematic review and meta‐analysis. JAMA Oncol. 2018;4(12):1721‐1728. 30242316 10.1001/jamaoncol.2018.3923 PMC6440712

[42] Ramos‐Casals M , Brahmer JR , Callahan MK , et al. Immune‐related adverse events of checkpoint inhibitors. Nat Rev Dis Prim. 2020;6(1):38. 32382051 10.1038/s41572-020-0160-6 PMC9728094

[43] Girault I , Adam J , Shen S , et al. A PD‐1/PD‐L1 proximity assay as a theranostic marker for PD‐1 blockade in patients with metastatic melanoma. Clin Cancer Res. 2022;28(3):518‐525. 34785583 10.1158/1078-0432.CCR-21-1229

[44] Rozeman EA , Hoefsmit EP , Reijers ILM , et al. Survival and biomarker analyses from the OpACIN‐neo and OpACIN neoadjuvant immunotherapy trials in stage III melanoma. Nat Med. 2021;27(2):256‐263. 33558721 10.1038/s41591-020-01211-7

[45] Hodi FS , Wolchok JD , Schadendorf D , et al. TMB and inflammatory gene expression associated with clinical outcomes following immunotherapy in advanced melanoma. Cancer Immunol Res. 2021;9(10):1202‐1213. 34389558 10.1158/2326-6066.CIR-20-0983 PMC9414280

[46] Xu J‐Y , Wei X‐L , Ren C , et al. Association of plasma Epstein‐Barr virus DNA with outcomes for patients with recurrent or metastatic nasopharyngeal carcinoma receiving anti–programmed cell death 1 immunotherapy. JAMA Netw Open. 2022;5(3):e220587. 35230439 10.1001/jamanetworkopen.2022.0587 PMC8889459

[47] Gao J , Navai N , Alhalabi O , et al. Neoadjuvant PD‐L1 plus CTLA‐4 blockade in patients with cisplatin‐ineligible operable high‐risk urothelial carcinoma. Nat Med. 2020;26(12):1845‐1851. 33046869 10.1038/s41591-020-1086-y PMC9768836

[48] Olson DJ , Eroglu Z , Brockstein B , et al. Pembrolizumab plus ipilimumab following anti‐PD‐1/L1 failure in melanoma. J Clin Oncol. 2021;39(24):2647‐2655. 33945288 10.1200/JCO.21.00079 PMC8376314

[49] Rangamuwa K , Leong T , Bozinovski S , et al. Increase in tumour PD‐L1 expression in non‐small cell lung cancer following bronchoscopic thermal vapour ablation. Transl Lung Cancer Res. 2021;10(6):2858‐2864. 34295683 10.21037/tlcr-21-76 PMC8264342

[50] Shi L , Chen L , Wu C , et al. PD‐1 blockade boosts radiofrequency ablation–elicited adaptive immune responses against tumor. Clin Cancer Res. 2016;22(5):1173‐1184. 26933175 10.1158/1078-0432.CCR-15-1352 PMC4780056

